# Microinstability of Major Joints in Movement Disorders: The Hidden Challenge

**DOI:** 10.7759/cureus.71449

**Published:** 2024-10-14

**Authors:** Rana Muhammad Anss Bin Qadir, Ahmad Hassan, Tanzeel Ur Rehman Buttar, Umar Bin Tariq, Wajeeha Kiran, M Hasaan Shahid

**Affiliations:** 1 Surgery, Queen’s Hospital, Romford, GBR; 2 Internal Medicine, NHS Lanarkshire Scotland, Glasgow, GBR; 3 General Medicine, Swansea Bay University Health Board, Swansea, GBR; 4 General Surgery, Southmead Hospital Bristol, North Bristol NHS Trust, Bristol, GBR; 5 Trauma and Orthopaedics, Morriston Hospital, Swansea, GBR; 6 Surgery, Glangwili General Hospital, Carmarthen, GBR

**Keywords:** biomechanics, joint stability, micro instability, movement disorders, neuromuscular control, proprioception, therapeutic interventions

## Abstract

Microinstability, characterized by subtle and often painful disturbances in joint stability, significantly impacts individuals engaged in activities requiring extensive ranges of motion, such as dancing or gymnastics, and is particularly prevalent in young female athletes. This condition, resulting from progressive microtrauma, architectural and functional abnormalities, or iatrogenic factors, is challenging to diagnose and often underreported. Understanding the biomechanical nuances of microinstability is essential for accurate diagnosis and effective management. Upper limb joints, including the shoulder, elbow, wrist, and hand, each exhibit unique anatomical and functional characteristics that contribute to their susceptibility to microinstability. Movement disorders, such as Parkinson's disease, essential tremor, dystonia, and cerebral palsy, exacerbate joint instability due to impaired proprioception, altered muscle tone, and uncoordinated muscle contractions. Effective diagnosis involves physical examination techniques and advanced imaging modalities. Therapeutic interventions encompass physical therapy, pharmacological treatments, surgical procedures, and assistive devices, tailored to enhance joint stability and improve the quality of life for affected individuals.

## Introduction and background

Microinstability refers to a subtle and often painful disturbance in joint stability that impairs function without causing visible dislocation or significant laxity. Unlike hyperlaxity, which is characterized by increased joint mobility without pain, microinstability is painful and typically results from progressive microtrauma rather than a single traumatic event [1]. It often affects individuals engaged in activities requiring extensive ranges of motion, such as dancing or gymnastics, and is therefore commonly seen in young female athletes. This condition is challenging to diagnose and may be underreported due to its subtle nature. Microinstability arises from architectural and functional abnormalities in the joint's supportive structures, including ligaments, tendons, and the joint capsule [2]. These abnormalities can result from bone deformities, deficiencies in the capsule-ligament complex, or muscle dysfunctions, compromising the joint's ability to maintain proper alignment and stability under stress. Additionally, iatrogenic factors, such as capsulotomy during hip arthroscopy, can also contribute to microinstability. Understanding these biomechanical nuances is essential for accurately diagnosing and effectively managing microinstability, particularly in at-risk populations [3].

The upper limb joints encompass the shoulder, elbow, wrist, and hand joints, each with unique anatomical and functional characteristics. The shoulder joint is a ball-and-socket joint, allowing extensive movement due to its shallow socket, which enhances flexibility at the expense of stability [4]. The elbow joint, primarily a hinge joint, permits flexion and extension, with the radial head and ulna enabling forearm rotation through a pivot mechanism. The wrist joint, an ellipsoidal or condyloid joint, along with the intercarpal joints, facilitates a range of hand movements, although the intercarpal joints have limited mobility [5]. The hand joints include the interphalangeal joints and basic hinge joints allowing the flexion and extension necessary for grasping and manipulating objects [6].

The aim of this study is to address microinstability in movement disorder patients and explore its implications for treatment and quality of life. Microinstability, characterized by excessive or abnormal movement within a joint, can significantly impact patients with movement disorders, exacerbating their symptoms and leading to increased pain and functional limitations. Studying microinstability helps in identifying the underlying causes of movement-related discomfort and unsteadiness, particularly in joints like the hip, which are essential for mobility. By exploring the mechanisms of microinstability, such as capsular laxity or connective tissue disorders, healthcare professionals can develop more targeted therapeutic strategies. Improved capsular management techniques, as highlighted in recent research, reduce the incidence of post-operative pain and the need for revision surgeries, thereby enhancing patient satisfaction and functional recovery. This focus is particularly relevant as it addresses a complex problem often seen in movement disorder patients who may experience higher rates of complications due to factors like generalized hypermobility. Enhanced diagnostic criteria and treatment protocols for microinstability can lead to better management of movement disorders, ultimately promoting stability and reducing pain for affected individuals.

## Review

The pathophysiology of microinstability in upper limb joints from a biomechanical perspective involves a variety of mechanisms leading to subtle yet clinically significant instability. These mechanisms often stem from ligamentous laxity, repetitive microtrauma, or muscle imbalances [7]. Ligamentous laxity, which may be congenital or acquired, weakens the supportive structures around joints such as the shoulder, allowing for excessive joint motion [8]. Repetitive microtrauma, common in activities requiring frequent overhead motions or heavy lifting, can progressively damage the joint capsule, ligaments, and labrum, compromising joint stability. Additionally, imbalances or weaknesses in the peri-articular musculature can fail to adequately support and stabilize the joint during movement, exacerbating instability. These biomechanical disruptions can lead to increased joint translation and abnormal motion, resulting in pain, inflammation, and potential damage to the articular surfaces. Effective treatment focuses on addressing these underlying biomechanical issues through targeted physical therapy and, in some cases, surgical intervention to restore joint stability and function [9].

Microinstability in the shoulder of overhead athletes significantly impacts joint function through biomechanical alterations that affect stability and movement dynamics. The repetitive stresses and extreme ranges of motion during throwing predispose the shoulder to microtraumatic injuries, compromising the integrity of static stabilizers like the capsulolabral complex and muscles [10]. This microinstability often results in anterior capsular laxity due to chronic tensile stress, leading to increased shoulder mobility [11]. Consequently, the anterior displacement of the humeral head can cause impingement of the rotator cuff and labrum against the glenoid rim, exacerbating conditions such as rotator cuff tendonitis and superior labral anterior to posterior (SLAP) lesions. These biomechanical disruptions not only diminish joint stability but also impair functional performance, necessitating targeted rehabilitation strategies to enhance dynamic stability, strengthen the rotator cuff and scapular muscles, and restore neuromuscular control to prevent further injury and optimize athletic performance [12].

Microinstability in upper limb joints involves intricate neurological contributions from both the central and peripheral nervous systems, crucial for maintaining joint stability and coordinated movements. The central nervous system orchestrates motor commands and coordinates muscle activation patterns necessary for precise joint movements. Any dysfunction in the central nervous system, such as stroke or cerebral palsy (CP), can disrupt these processes, leading to impaired intra-limb coordination and compromised joint stability [13]. Additionally, the peripheral nervous system plays a vital role by transmitting sensory information from the joints to the central nervous system, providing feedback on joint position and movement. Damage or dysfunction in peripheral nerves can impair proprioception and kinesthetic awareness, further exacerbating microinstability in upper limb joints. Therefore, a comprehensive understanding of both central and peripheral neurological contributions is essential in developing effective rehabilitation strategies to restore optimal neuromuscular control and functional stability in patients with microinstability [14].

The interaction between neural control and joint stability plays a pivotal role in the pathophysiology of microinstability in upper limb joints. Neural mechanisms involved in movement control extend beyond the initiation and execution of movements to include the crucial ability to halt movements precisely and stabilize the limb. This stopping process relies on sophisticated neural circuits that integrate sensory feedback with motor commands to modulate muscle activity and adjust limb impedance dynamically [15]. For instance, during the deceleration phase of a movement, coordinated activation of agonist and antagonist muscles ensures smooth and controlled stopping, preventing overshoot or instability at the intended endpoint. Damage or dysfunction in these neural circuits, as seen in conditions like stroke or CP, can disrupt this delicate balance of muscle activation patterns, leading to compromised joint stability and contributing to microinstability in joints. Understanding these neural contributions is essential for developing targeted interventions aimed at restoring optimal neuromuscular function and enhancing joint stability in clinical populations [16].

Microinstability in upper limb joints, particularly in overhead-throwing athletes, involves a delicate interplay between neural control and joint stability. The dynamic stabilization of the shoulder relies heavily on neuromuscular control, which involves the coordinated activity of muscles to maintain joint integrity during the high forces and extreme ranges of motion typical in throwing [10]. Effective neuromuscular control is dependent on accurate proprioceptive feedback - the sensory information about joint position and movement - which enables timely and appropriate muscular responses. Repetitive stress and microtraumas from throwing can impair proprioceptive input, leading to suboptimal motor control and compromised joint stability. This can result in increased laxity and microinstability, particularly in the anterior capsule of the shoulder [17]. Studies have shown that excessive external rotation during the throwing motion can elongate the anterior band of the inferior glenohumeral ligament, exacerbating anterior and inferior joint translation. Therefore, a comprehensive rehabilitation program for these athletes must prioritize enhancing proprioception and neuromuscular control to stabilize the joint dynamically and prevent further injury [18].

Parkinson’s disease (PD), a prevalent neurodegenerative movement disorder, is marked by symptoms such as resting tremor, rigidity, bradykinesia, and postural instability, primarily resulting from dopamine depletion and the degeneration of dopaminergic neurons. These motor symptoms significantly affect the upper limb's ability to maintain stability, contributing to microinstability [19]. Approximately 6.2 million people worldwide are affected by PD, with its progression often linked to mitochondrial dysfunction and oxidative stress. The muscle rigidity and tremors characteristic of PD place additional stress on the joints, exacerbating instability. The prevalence of microinstability in PD patients is considerable, driven by impaired proprioception and altered muscle tone, which are critical for joint stability. Moreover, the degeneration of neuronal circuits beyond the dopaminergic system and the systemic pathology, including gastrointestinal involvement and neuroinflammation, further complicate the stability of upper limb joints. Addressing these multifaceted issues through advanced therapeutic strategies targeting mitochondrial dysfunction and neuroinflammation is crucial in managing microinstability in PD patients [20,21].

Essential tremor (ET) is the most prevalent neurologic cause of postural or action tremor, typically presenting as a bilateral tremor in the hands at a frequency of 6 to 12 Hz. This involuntary rhythmic movement significantly impacts joint stability in the upper limbs. The continuous tremor leads to persistent, involuntary contractions of muscles around the joints, which disrupts the fine balance required for stable joint function. This instability can hinder the performance of precise tasks such as writing or using utensils, as the joints cannot maintain a steady position. Moreover, the compensatory mechanisms used by patients, such as excessive muscle co-contraction, may further exacerbate joint instability by increasing stiffness and reducing the range of motion [22,23]. Consequently, ET can contribute to overuse injuries and chronic musculoskeletal issues, making management strategies critical to improving joint stability and overall quality of life for affected individuals. These strategies may include pharmacological interventions, physical therapy, and in severe cases, surgical options like deep brain stimulation to reduce tremor severity and enhance joint stability [22,24].

Dystonia is characterized by sustained or intermittent muscle contractions leading to abnormal movements or postures. The microinstability in dystonic movements arises from the uncoordinated and involuntary muscle contractions that create excessive and unpredictable forces on the joints. This instability is exacerbated by the inability to maintain stable muscle tone and control, resulting in erratic and forceful movements that disrupt the normal joint mechanics. The unpredictable nature of dystonic contractions, often triggered by voluntary movements or external stimuli, leads to an imbalance in the muscle forces around the joint, causing repetitive strain and microtrauma to the tissues. Over time, this can result in joint degeneration, chronic pain, and functional impairment [25]. The pathophysiology of dystonia involves abnormalities in the basal ganglia and other neural circuits that regulate motor control, contributing to the loss of inhibition and aberrant motor signals [26]. Understanding these mechanisms is crucial for developing targeted therapies to manage microinstability and improve the quality of life for individuals with dystonia [25].

CP is a neurological disorder characterized by abnormal muscle tone, posture, and movement, often resulting in significant upper limb joint instability. Children and adults with CP commonly experience spasticity, muscle weakness, and lack of coordination, contributing to joint instability [27]. These symptoms can vary in severity depending on the type of CP, such as spastic hemiplegia, spastic diplegia, spastic quadriplegia, and dyskinetic forms [28]. The increased muscle tone, or spasticity, leads to stiffness and limited range of motion in the joints, while muscle weakness impairs the ability to stabilize joints effectively, making them prone to subluxations and dislocations. Coordination difficulties further exacerbate these challenges, affecting fine motor skills and the ability to perform precise movements. This instability not only impacts functional capabilities but also increases the risk of chronic pain and deformities over time [29]. Interventions such as physical therapy, occupational therapy, and sometimes surgical procedures are essential in managing these issues, aiming to improve joint stability, enhance motor function, and ultimately improve the quality of life for individuals with CP [28].

The clinical evaluation of microinstability involves employing specific physical examination techniques aimed at detecting subtle abnormalities indicative of this condition. One such approach is the abduction-hyperextension-external rotation (AB-HEER) test, which has demonstrated high accuracy in diagnosing hip instability. This test is conducted with the patient in the lateral decubitus position, where the affected hip is abducted, extended, and externally rotated, while an anteriorly directed force is applied to the posterior greater trochanter. A positive AB-HEER test result is characterized by the reproduction of anterior hip pain, indicating potential microinstability. This maneuver showed a sensitivity of 80.6% and a specificity of 89.4% in a cohort study assessing its diagnostic accuracy. Complementary to the AB-HEER test, the prone instability test and hyperextension-external rotation (HEER) test were also evaluated, each revealing varying levels of sensitivity and specificity. These physical examination maneuvers are crucial tools for clinicians striving to diagnose hip microinstability, a condition increasingly recognized for its impact on young patients and athletes experiencing persistent hip-related disability and pain [30,31].

The utilization of MRI, CT, and ultrasound in evaluating joint instability is critical for diagnosing and managing musculoskeletal disorders. MRI offers detailed images of soft tissues, such as ligaments and tendons, providing insights into joint abnormalities like tears and dislocations. Its non-invasive nature and high-resolution capabilities make MRI particularly effective for assessing joint instability. CT scans, on the other hand, excel in capturing bone structures and are valuable for identifying fractures and bone dislocations contributing to joint instability [32]. Additionally, ultrasound imaging serves as a versatile tool for real-time assessment of joint movement and can guide diagnostic and therapeutic procedures [33]. These imaging modalities collectively enable clinicians to accurately diagnose joint instability, facilitating prompt treatment decisions and improving patient outcomes [34].

Kinematic and kinetic analysis methods are pivotal in biomechanics for evaluating human movement dynamics through precise measurements of body segment positions, velocities, and accelerations. These assessments are crucial for understanding how forces act on the body during physical activities such as sports or rehabilitation exercises. Kinematic analysis involves using technologies like motion capture systems and high-speed cameras to track and measure the movement of specific body parts in three-dimensional space. This data provides detailed insights into the mechanics of movements like walking or running, aiding in diagnosing movement disorders and optimizing performance [35,36]. Concurrently, the kinetic analysis focuses on quantifying the forces and torques generated by muscles and external loads, revealing the biomechanical forces contributing to joint stability and injury risk [37]. By integrating kinematic and kinetic analysis methods, clinicians and researchers can effectively diagnose movement impairments, monitor rehabilitation progress, and tailor interventions to enhance movement efficiency and mitigate injury risks in various patient populations [36].

Therapeutic interventions for micro-instability of joints in patients with movement disorders can be significantly enhanced through targeted physical therapy and rehabilitation [38]. An effective approach includes a combination of endurance, resistance, flexibility, balance, and multicomponent exercises tailored to individual needs. Endurance training, characterized by rhythmic, sustained activity, improves cardiovascular and muscular endurance, which supports joint stability and reduces the risk of falls. Resistance exercise focuses on strengthening the muscles surrounding the joints, improving their ability to stabilize and support movement. Flexibility exercises enhance the range of motion and reduce joint stiffness, while balance and multicomponent training address motor control and proprioception [39,40]. A personalized exercise program that incorporates these elements, adjusted for the patient’s fitness level, personal factors, and specific needs, can lead to improved joint stability and overall functional outcomes, thereby enhancing the quality of life for individuals with movement disorders [41].

Pharmacological treatments for managing micro-instability of joints in patients with movement disorders aim to address both muscle tone and joint stability, which can be significantly affected by spasticity and muscle stiffness [42]. These conditions are prevalent in individuals suffering from neurological injuries such as stroke [43], CP, spinal cord injury, and multiple sclerosis. Muscle stiffness, characterized by increased resistance to passive stretch and persistent spasticity, can contribute to severe disability and slowed recovery. To mitigate these effects, various medications are employed. Antispasticity agents like baclofen and tizanidine work by decreasing muscle tone, thus improving joint stability and facilitating more controlled movements. Muscle relaxants such as dantrolene also help in reducing excessive muscle rigidity. Botulinum toxin injections can provide targeted relief by reducing localized muscle over-activity, though they may not address all aspects of muscle stiffness, particularly those linked to non-neural changes in muscle and connective tissue. Additionally, CNS depressants, including benzodiazepines, can aid in reducing spasticity but may induce side effects like muscle weakness and fatigue. By combining these pharmacological strategies, clinicians aim to improve functional outcomes and enhance the quality of life for patients with movement disorders [43,44].

Surgical intervention for the treatment of microinstability of joints, particularly in movement disorders, is considered when conservative treatments fail to alleviate symptoms. Indications for surgical intervention include persistent pain, recurrent instability, and significant functional impairment despite non-surgical management [45,46]. Various surgical techniques are available to address microinstability, each tailored to the specific joint and underlying pathology. Capsular repair is a common procedure that has shown improved outcomes, particularly in patients with residual capsular defects following initial surgeries, such as those with femoroacetabular impingement. Capsular plication, another effective technique, aims to tighten the joint capsule to enhance stability and is often employed when there is a diagnosis of hyperlaxity or previously failed hip arthroscopies. For more severe cases or when capsular structures are extensively damaged, capsular reconstruction using allografts, such as the iliotibial band, provides a robust option to restore joint stability [47]. Additionally, ligamentum teres reconstruction is indicated in specific cases of hip instability, particularly in patients with connective tissue disorders like Ehlers-Danlos syndrome [48]. These surgical options are crucial in restoring joint stability, improving pain, and enhancing functional outcomes, especially in complex cases where non-surgical treatments are inadequate [49].

The treatment of microinstability of joints in movement disorders often involves the strategic use of assistive devices, such as orthoses, to enhance joint stability. Modern advancements in assistive technology have led to the development of sophisticated orthotic devices that offer targeted support to joints, effectively addressing issues related to instability [49,50]. For instance, recent innovations include active and passive exoskeletons designed to assist in critical movements of the ankle, knee and hip joints, which are pivotal for maintaining stability during locomotion. These devices work by providing assistive torque that compensates for weakened or unstable joint functions. The effectiveness of such devices is closely tied to their design, which must balance the benefits of external assistance with potential drawbacks, such as added mass and kinematic constraints [51,52]. By leveraging an understanding of human anatomy and biomechanics, researchers are optimizing these devices to reduce metabolic costs and improve overall joint stability, ultimately enhancing the mobility and quality of life for individuals with movement disorders [53].

## Conclusions

Microinstability of major joints poses a significant challenge, especially in individuals with movement disorders. The intricate interplay of biomechanical and neurological factors contributes to this condition, which can severely impact joint function and overall quality of life. Comprehensive diagnostic approaches, including physical examination techniques and advanced imaging modalities, are crucial for accurately identifying microinstability. Therapeutic strategies, encompassing targeted physical therapy, pharmacological treatments, surgical interventions, and the use of assistive devices, are essential in managing this condition. By addressing the underlying biomechanical and neurological contributors, these interventions aim to restore joint stability, reduce pain, and enhance functional outcomes. Continued research and advancements in treatment modalities are vital for improving the management of microinstability in patients with movement disorders, ultimately promoting better quality of life and functional independence.
